# Transcriptional Profiling of ESTs from the Biocontrol Fungus *Chaetomium cupreum*

**DOI:** 10.1100/2012/340565

**Published:** 2012-02-01

**Authors:** Haiyan Zhang, Min Li

**Affiliations:** ^1^College of Life Science, Henan University, Kaifeng 475001, China; ^2^Institute of Bioengineering, Henan University, Kaifeng 475001, China; ^3^Faculty of Chemistry Biology and Material Sciences, East China Institute of Technology, Jiangxi, Fuzhou 344000, China

## Abstract

Comparative analysis was applied to two cDNA/ESTs libraries (C1 and C2) from *Chaetomium cupreum*. A total of 5538 ESTs were sequenced and assembled into 2162 unigenes including 585 contigs and 1577 singletons. BlastX analysis enabled the identification of 1211 unigenes with similarities to sequences in the public databases. MFS monosaccharide transporter was found as the gene expressed at the highest level in library C2, but no expression in C1. The majority of unigenes were library specific. Comparative analysis of the ESTs further revealed the difference of *C. cupreum* in gene expression and metabolic pathways between libraries. Two different sequences similar to the 48-KDa endochitinase and 46-KDa endochitinase were identified in libraries C1 and C2, respectively.

## 1. Introduction

One of the reasons for environmental disorder is that modern agriculture is an ecologically unbalanced system which has been destroyed by chemical fungicides. Biocontrol is highly interesting alternative method of chemical plant disease control. *C. cupreum *Ames is an ascomycete fungus with considerable biocontrol potential to plant fungal pathogens, especially several notorious examples belonging to the genera of *Pythium*, *Rhizoctonia*, and *Pyricularia *[1]. In Thailand and China, its biological products have been applied in agricultural disease management [1, 2]; nevertheless, the genetic basis of the defense mechanisms of *C. cupreum* is not well understood thus inhibiting its application. 

ESTs analysis has been proven to be an efficient and valuable tool in obtaining coding gene information, understanding the pathways involved in a given physiological or environmental stimulus [3, 4]. To date, several ESTs studies have been carried out on fungal biocontrol agents, especially to species of *Trichoderma*. For example, analysis of 8,710 ESTs of* T. harzianum* CECT 2413 from eight cDNA libraries including those simulating mycoparasitism [5] and of ESTs from four different *Trichoderma* strains grown under conditions related to biocontrol [6].

In the present study, we performed comparative analysis of two cDNA/ESTs libraries from *C. cupreum*. An obvious difference in gene expression and metabolic pathways were detected between libraries. This research contributes to elucidating further the mycoparasitisic molecular mechanisms involved in *C. cupreum* and will help to develop novel strategies in fungal disease management.

## 2. Materials and Methods

### 2.1. Fungal Strains and Culture Conditions

The *C. cupreum *isolate was kindly provided by King Mongkut's Institute of Technology of Ladkrabang, Thailand.* Rhizoctonia solani *was stored in our laboratory.

C1 was constructed from mycelia of *C. cupreum* grown on potato dextrose agar (PDA) culture. Library C2 was constructed with mycelia of *Rhizoctonia solani* cell wall (RsCW) as the carbon source, this was designed to resemble the phases of interaction with* R. solani*. For C1 library construction, mycelia of *C. cupreum* were cultured on potato dextrose (PD) medium for 60 h at 27°C and 150 rpm. The biomass was harvested and stored at −80°C until use. For C2 library construction, mycelia were initially grown in PD medium with shaking at 27°C and 150 rpm for 24 h then transferred to SM medium [2.8 g (NH_4_)_2_SO_4_ l^−1^; 0.6 Urea g l^−1^; 4 g KH_2_PO_4_ l^−1^; 0.6 g CaCl_2_ *·*2H_2_O l^−1^; 0.2 g MgSO_4_ l^−1^; 0.01 g FeSO_4_ *·*7H_2_O l^−1^; 0.0028 g ZnSO_4_ *·*H_2_O l^−1^; 0.0032 g CoCl_2_ *·*6H_2_O l^−1^; 5 g RsCW l^−1^] and incubated for a further 36 h at 27°C and 150 rpm. 

### 2.2. Construction of the cDNA Libraries and DNA Sequencing

Total RNA was extracted using Trizol reagent from mycelia of *C. cupreum*. Polyadenylated RNA was purified using an Oligotex mRNA Kit (Qiagen). The course of cDNA library construction followed the procedures of Zhang as described before [7]. Unidirectional cDNA libraries were constructed using the pBluescript II plasmid system. Fragments of cDNA clones were sequenced using a T3 primer from the 5′ end with MegaBase1000 DNA sequencer. 

### 2.3. Data Processing and Bioinformatics Analysis

Vector sequences, sequences shorter than 100 bp and containing more than 5% ambiguous bases, were discarded using the Crossmatch program. High-quality sequences were assembled using Phrap (http://www.phrap.org/) and accuracy of contigs was confirmed with Consed [8]. All unigenes were compared against public nonredundant (nr) protein databases using a BlastX search. According to KEGG (Kyoto Encyclopedia of Genes and Genomes) [9], unigenes were assigned to different metabolic pathways with the same criterion as described by Zhang [7]. All high-quality ESTs were submitted to the GenBank database under accession numbers *DV544375-DV548659*.

## 3. Results

### 3.1. ESTs Clustering and Function Assignment

A total of 5,538 cDNA clones with an insert size of more than 700 bp were selected for sequencing, resulting in 4285 ESTs (3066 from C1 and 1219 from C2) after removing sequences representing ribosomal, vector, and low-quality sequences. Minimum, average, and maximum lengths of ESTs were 102, 518, and 795 bp, respectively, with a large fraction falling between 500 and 700 bp (2110 from C1 and 795 from C2) in both libraries.

Using the Phrap and Consed programs, ESTs from both libraries were arranged into 585 contigs and 1577 singletons, giving a total of 2162 unigenes. Each unigene was subjected to analysis against homologous sequences in public protein databases using the BlastX algorithm. Approximately 1211 (56%) of the unigenes were assigned a function with an *E*-value of 10^−5^ or lower. The remaining 951 clones had no high homology to genes of known function. A total of 1138 (52.6%) and 691 (32%) unigenes were unique and only expressed in C1 or C2, respectively.

### 3.2. Exploration of Highly Expressed ESTs

Contigs containing 4 or more ESTs from each library are listed in Table 1. Of the 26 clusters, more than one third were (10/26) expressed only in the C1 library, 2 only in the C2 library, and half (14/29) in both but at a different level.

Glyceraldehyde-3-phosphate dehydrogenase was the most highly expressed transcript (109 ESTs) in the C1 library, occurring four times more than in the C2 library. The most highly represented transcripts in the C2 library coded for a putatively major facilitator superfamily (MFS) monosaccharide transporter; no such expression was observed in library C1. The expression of coproporphyrinogen oxidase, thiazole biosynthetic enzyme, glutamine synthetase, ATP citrate lyase, and aspartate aminotransferase were higher in the C1 library. It should be noted that many hits similar to predicted or unknown function proteins were detected in libraries. They are ideal candidates for future study.

### 3.3. Metabolic Pathways Analysis

Pathways analysis of KEGG was carried out using genes homologous to known functional sequences. A total of 65 and 61 different metabolic pathways were found in the C1 and C2 libraries, respectively. These results show evident difference in metabolic pathways between the libraries (Table 2).

Glycolysis/gluconeogenesis was the most represented pathway within each library. The second and third most enriched functional pathways in the C1 library were porphyrin and chlorophyll metabolism, which involved 180 ESTs (17.1%), and the citrate cycle, which involved 47 ESTs (4.5%). In the C2 library, the respective pathways were peptideprotein biosynthesis and d-arginine and ornithine metabolism.

It should be noted that the types of genes involved in the same metabolic pathways were greatly different between libraries. For instance, in the glycolysis/gluconeogenesis pathway, the enzymes in the C1 library were enolase, glyceraldehyde 3-phosphate dehydrogenase, fructose 1,6-biphosphate aldolase, and pyruvate decarboxylase, which were assigned to glycolysis; however, in the C2 library, they were fructose-1,6-bisphosphatase, pyruvate carboxylase, and phosphoenolpyruvate carboxykinase, which were assigned to gluconeogenesis. Because glucose is a very important source of nutrition, we speculate that the upregulated genes related to gluconeogenesis observed in the C2 library may be necessary for mycoparasitism, that is, maintenance of fast cell growth rate in response to the competition with the plant fungal pathogen.

### 3.4. Genes Induced by the Mycoparasitic Process

**Figure 1:**
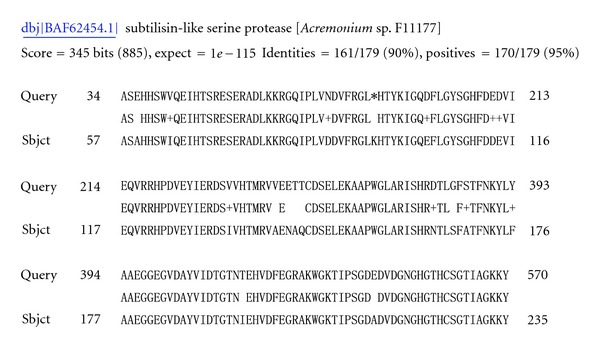
DV546294_shared 90% identity with serine proteases of *Acremonium* sp.

**Figure 2:**
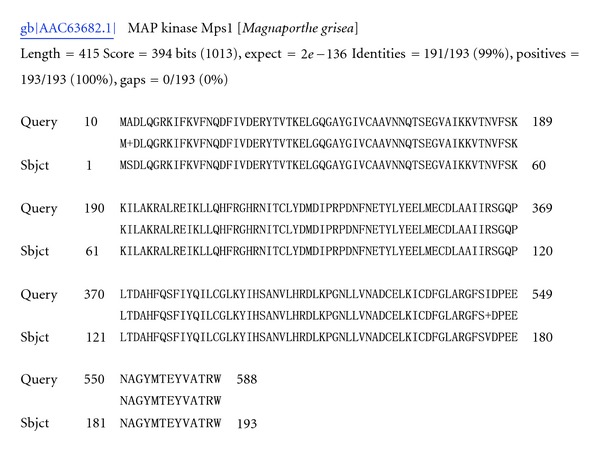
DV548513 shared 98% identity with MAP kinase of *Gibberella intermedia*.

Differences were observed in gene groups associated with degradation of the cell wall, proteolysis, and toxins production. Seven contigs were presented in both libraries, four were specific to library C1, and eight to library C2 (Table 3), the latter appearing to be induced by the mycoparasitic process directly. Two sequences similar to the 48-KDa endochitinase (GenBank accession nos. *DV546055, DV544732, *and* DV544989*) of *Aspergillus nidulans *and 46-KDa endochitinase (*DV547883 *and* DV547485*) of *Hypocrea virens *were identified in libraries C1 and C2, respectively. Four ESTs from library C1 (*DV546459*, *DV546294*, *DV544423*, and* DV546484*) and 1 (*DV548260*) from library C2 shared similarity with serine proteases (Figure 1). One-gene homologue of MAP kinase A (*DV548513*) was identified in library C2 only (Figure 2).

## 4. Discussion

It has been demonstrated that the cell wall of the fungal pathogen can simulate some aspects of the mycoparasitic interactions between biocontrol fungi and its targets [10].

Only a limited amount of overlap (333 unigenes) was observed in both libraries. A total of 1138 and 691 unigenes were unique and only expressed in C1 or C2, respectively. The lack of significant overlap between the individual libraries also suggests a high level of flexibility at the level of gene expression under the examined conditions, some of which may reflect particular requirements for phases of mycoparasitism.

The analysis of the frequency of specific ESTs that form individual contigs can give information about the expression levels of particular genes under different experimental conditions [11]. The most abundant transcripts in library C2 but no expression in C1 were MFS monosaccharide transporters. MFS transporters transport uni-, sym-, and antiporters of sugars, peptides, drugs, and organic and inorganic ions with 12 or 14 transmembrane spanners [12]. In the present study, the high proportion of ESTs expressing a homology to MFS monosaccharide transporters implies that they may be responsible for transport of monosaccharides derived from the degradation of RsCW. This was not consistent with the results of a study of *T. harzianum* CECT 2413 [13], in which abundant expression of peptide transporter 2 (PTR2) was found in a cDNA library of *T. harzianum* CECT 2413 when interacted directly with *Botrytis cinerea*. However, only one EST similar to PTR2 (*DV547977*) was detected in library C2. We speculate that this may have been caused by the different cultivation times of the two fungi.

Comparison analysis illustrated variations in the proportions of different pathways. Metabolic pathways of ubiquinone biosynthesis; electron transport and oxidative phosphorylation; purine metabolism; pyrimidine metabolism; alanine and aspartate metabolism; valine, leucine, and isoleucine biosynthesis; porphyrin and chlorophyll metabolism were proportionately more represented in library C1. In contrast, pentose and glucuronate interconversions; fructose and mannose metabolism; galactose metabolism; androgen and estrogen metabolism; glycine, serine, and threonine metabolism; valine, leucine, and isoleucine degradation; arginine and proline metabolism; histidine metabolism; tryptophan metabolism; d-arginine and ornithine metabolism; glycerolipid metabolism were overrepresented in library C2. Metabolic pathways of sterol, vitamin K, vitamin E, carotenoids biosynthesis; sulfur metabolism: reduction and fixation; DNA polymerase; cytochrome C oxidase were only observed in library C1, while those of fatty acid biosynthesis (path 2), styrene degradation, and Vitamine B6 metabolism were only observed in library C2.

The results showed that genes related to mycoparasitism were differentially expressed. Two different sequences similar to the 48-KDa endochitinase and 46-KDa endochitinase were identified in libraries C1 and C2, respectively. Since library C1 was obtained from cultivation on PDA medium, the 48 KDa endochitinase homolog might play a role in the dissolution and formation of the cell wall of* C. cupreum*. Similarly, because library C2 was constructed under conditions associated with mycoparasitism, the 46 KDa endochitinase homolog is expected to be involved in cell wall degradation of the fungal pathogen during the mycoparasitic process.

**Figure 3:**
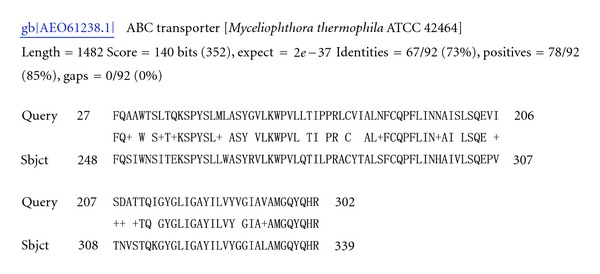
DV548480 shared 73% identity with ABC transporter of *Myceliophthora thermophila* ATCC 42464.

The conditions used for construction of library C2 were aimed at *in vitro* simulation of the mycoparasitic process, which is triggered by the recognition of the structural character of the pathogenic fungal cell wall. As a result, the genes involved in signal transduction pathways of mycoparasitism were acquired. Examples include homologue of gene encoding an ABC transporter (ATP-binding cassette transporter, *DV548480*, Figure 3) and MAP-kinase A (Tmk1 of *T. atroviride*). Four ESTs have sequence homology to an ABC transporter, it was also observed previously in other fungal pathogens (*Gibberella pulicaris* and* Sclerotinia sclerotiorum*) Mehrabi et al. [14] as potential pathogenicity factors responsible for tolerance to phytoalexins or a pathogenicity factor for the host Fleissner et al. [15] and Li et al. [16]. Studies on signal transduction pathways from *Trichoderma *strains revealed the involvement of MAP-kinases in the mycoparasitic interaction, including production of hydrolytic enzymes such as chitinases and secretion of antibiotic substances [17].

In this study, we sequenced and analyzed two independent cDNA libraries, providing the first comparative analysis of the transcriptome of* C. cupreum* under different conditions. The findings provide an entry point for understanding further the molecular mechanisms of this fungus and will also help to advance our efforts in developing novel strategies for biocontrol of fungal diseases.

## Figures and Tables

**Table 1:** The difference of high-redundancy genes expression between cDNA libraries from *C. cupreum*.

Contig no.	Annotation	C1		C2	
		ESTs	%	ESTs	%
Contig525	Glyceraldehyde-3-phosphate dehydrogenase	109	7.41	14	1.69
Contig531	Coproporphyrinogen oxidase	72	4.89	5	0.6
Contig30	Predicted protein	56	3.81	0	0
Contig433	C-4 sterol methyl oxidase	54	3.67	0	0
Contig313	Predicted protein	36	2.45	0	0
Contig2	Pyruvate decarboxylase	27	1.84	0	0
Contig39	Predicted protein	23	1.56	0	0
Contig507	Xylulose-5-phosphate phosphoketolase	21	1.43	3	0.36
Contig 152	Predicted protein	20	1.36	16	1.93
Contig523	EF1-alpha translation elongation factor	19	1.29	12	1.45
Contig42	Thiazole biosynthetic enzyme	19	1.29	2	0.24
Contig520	Actin	17	1.16	4	0.48
Contig495	Glutamine synthetase	17	1.16	1	0.12
Contig517	Aspartic protease	16	1.09	3	0.36
Contig317	Ammonium transporter	16	1.09	0	0
Contig419	ATP synthase protein	16	1.09	0	0
Contig4	Alcohol dehydrogenase	15	1.02	4	0.48
Contig474	*β*-1,3-exoglucanase	14	0.95	2	0.24
Contig494	ATP citrate lyase	14	0.95	1	0.12
Contig412	Histone H2B	13	0.88	0	0
Contig298	Pyruvate kinase	13	0.88	0	0
Contig310	Aspartate aminotransferase	11	0.75	1	0.12
Contig408	Heat shock protein 30	11	0.75	0	0
Contig489	ADP-ATP translocase	11	0.75	8	0.97
Contig165	MFS monosaccharide transporter	0	0	20	2.42
Contig162	Phosphoenolpyruvate carboxykinase	0	0	12	1.45

Relative values (%): ESTs numbers/all ESTs numbers in C1 or C2 library.

**Table 2:** The metabolic pathways difference between cDNA libraries from *C. cupreum*.

Map no.	Pathways	ESTs in C1	ESTs in C2
00010	Glycolysis/gluconeogenesis	188	31
00020	Citrate cycle	47	9
00030	Pentose phosphate cycle	35	6
00040	Pentose and glucuronate interconversions	6	4
00051	Fructose and mannose metabolism	4	10
00052	Galactose metabolism	7	7
00053	Ascorbate and aldarate metabolism	5	5
00061	Fatty acid biosynthesis (path 1)	3	1
00062	Fatty acid biosynthesis (path 2)	0	1
00071	Fatty acid metabolism	24	12
00100	Sterol, vitamin K, vitamin E, carotenoids biosynthesis	1	0
00120	Bile acid biosynthesis	19	12
00130	Ubiquinone biosynthesis	14	3
00150	Androgen and estrogen metabolism	4	12
00190	Electron transport and oxidative phosphorylation	34	6
00220	Urea cycle and metabolism of amino groups	10	0
00230	Purine metabolism	41	1
00240	Pyrimidine metabolism	6	1
00251	Glutamate metabolism	24	4
00252	Alanine and aspartate metabolism	14	1
00260	Glycine, serine, and threonine metabolism	2	8
00271	Methionine metabolism	12	5
00272	Cysteine metabolism	6	1
00280	Valine, leucine, and isoleucine degradation	6	6
00290	Valine, leucine, and isoleucine biosynthesis	12	1
00300	Lysine biosynthesis	4	1
00310	Lysine degradation	6	5
00330	Arginine and proline metabolism	15	6
00340	Lysine degradation	4	4
00350	Tyrosine metabolism	25	9
00360	Phenylalanine metabolism	9	6
00361	Gamma-hexachlorocyclohexane degradation	5	6
00380	Tryptophan metabolism	22	14
00400	Phenylalanine, tyrosine, and tryptophan biosynthesis	13	13
00410	*B*-alanine metabolism	5	5
00440	Aminophosphonate metabolism	4	1
00450	Selenoamino acid metabolism	14	5
00472	D-arginine and ornithine metabolism	2	15
00480	Glutathione metabolism	8	2
00500	Starch and sucrose metabolism	16	11
00520	Nucleotide sugars metabolism	7	4
00530	Aminosugars metabolism	7	5
00550	Peptide protein biosynthesis	17	0
00561	Glycerolipid metabolism	5	9
00562	Inositol phosphate metabolism	4	2
00600	Sphingoglycolipid metabolism	4	3
00620	Pyruvate metabolism	35	10
00626	Nitrobenzene degradation	1	1
00630	Glyoxylate and dicarboxylate metabolism	9	4
00640	Propanoate metabolism	9	6
00643	Styrene degradation	0	1
00650	Butanoate metabolism	13	6
00670	One-carbon pool by folate	6	3
00680	Methane metabolism	35	3
00740	Riboflavin metabolism	5	5
00750	Vitamine B6 metabolism	0	1
00760	Nicotinate and nicotinamide metabolism	4	3
00770	Pantothenate and CoA biosynthesis	9	1
00790	Folate biosynthesis	4	2
00860	Porphyrin and chlorophyll metabolism	180	6
00920	Sulfur metabolism: reduction and fixation	2	0
00950	Alkaloid biosynthesis I	7	0
00970	Aminoacyl-tRNA biosynthesis	11	2
03020	RNA polymerase	3	1
03030	DNA polymerase	4	0
03130	Cytochrome C oxidase	1	0
03140	Cytochrome C reductase	10	4
03150	Succinate dehydrogenase	3	1

**Table 3:** The different expression of genes related to biocontrol between cDNA libraries from *C. cupreum*.

Annotation	C1	C2
	ESTs	ESTs
48 KDa endochitinase	3	0
46 KDa endochitinase	0	2
*β*-N-acetylglucosaminidase	0	1
*β*-1,3-exoglucanase	22	2
*β*-1,3-endoglucanase	1	0
*β*-glucosidase D	1	1
*β*-glucosidase 5	6	0
*β*-glucosidase 6	1	0
Serine protease	4	1
Aspartic proteinase	20	5
Immune antigen 1	0	1
C-8 sterol isomerase	2	2
Sterol C-22 desaturase	1	1
Sterol-C5-desaturase	2	2
*β*-endo-1,4-xylanase	0	3
Peptide transporter 2	0	1
MAP kinase A	0	1
ABC transporters	0	1
